# Pediatric residents’ and attending physicians’ perspectives on the ethical challenges of end of life care in children

**Published:** 2018-12-30

**Authors:** Alireza Ebrahimi, Sedigheh Ebrahimi

**Affiliations:** 1 *Student of Medicine,* *Student Research Committee, Shiraz University of Medical Sciences, Shiraz, Iran.*; 2 *Associate Professor, Department of Medical Ethics, Shiraz University of Medical Sciences, Shiraz, Iran.*

**Keywords:** *Ethics*, *Medical residents*, *Palliative care*, *Pediatrics*

## Abstract

One issue that has received less attention in present health care protocols is pediatric palliative care (PPC), which is an approach to care starting with the diagnosis of life-threatening diseases in children. It embraces physical, emotional and spiritual elements. Ethical issues are major concerns in today’s pediatric health care guidelines and must be considered by residents and attending physicians in this field.

The present study was conducted in Namazi Teaching Hospital, Shiraz, Iran**. **Forty-eight out of 92 pediatricians were enrolled in this research, including 8 attendings, 6 fellows, and 34 residents. The study questionnaire consisted of 66 items. It was built based on previous reliable and validated questionnaire; also the calculated Cranach’s alpha was 0.815. Data were analyzed and presented by mean SD and percentage.

While seventy-five percent of the participants reported involvement in pediatric palliative care, fifty-six percent did not acknowledge any information about the subject. More than half of the participants perceived the pediatric palliative care services in Namazi Hospital as somewhat or completely satisfactory. Furthermore, thirty-five percent of the applicants stated that they encounter an ethical problem with regard to PPC once a week.

There are many challenges to providing decent palliative care for children, including symptom controlling, shifting to end of life care, background dissimilarities of patients, financial restrictions, and acceptance of death. Our applicants believed that offering psycho-spiritual support was the most important challenge in PPC. However, further investigations are needed to determine other requirements for providing a comprehensive guideline on PPC.

## Introduction

Palliative care (PC) has been internationally acknowledged as a human right and a public health issue, since it involves themes such as dignity of individuals, universality and nondiscrimination (1). As announced in article no. 25.1 of the Universal Declaration of Human Rights, “Everyone has the right to a standard of living adequate for the health of himself and his family, including food, clothing, housing and medical care and necessary social services….” (2). “Attention and care for chronically and terminally ill persons, sparing them avoidable pain and enabling them to die with dignity” has also been mentioned in General Comment no. 14 (2). World Health Organization (WHO) defines palliative care as active care for patients who have no curative alternative, and the principal points include control of pain and other symptoms such as psychological, social and spiritual breakdowns in order to improve the quality of life (QoL) (3, 4). 

Pediatric Palliative Care (PPC) could be defined as measures taken to manage children who are suffering from terminal diseases, and it should be applied when cure cannot be achieved by other treatments (5, 6). PPC has physical, emotional, social and spiritual aspects and is aimed at helping patients and their families improve their QoL throughout death and loss (5, 6). Educating the patients and their families, social support, and involving the affected parties in discussions about planning PPC should all be handled in an open and transparent way (5, 7). The emerging paradigm of PPC embraces the concept of applying PPC codes at the primitive stages of life threatening diseases and creating a multidisciplinary system of support around the children and their families, and can be provided in different settings such as the hospital or hospice care unit, school and home (8, 9). 

Usually, children - especially young children - cannot engage in the decision-making process; however, they often know they are dying and they may have treatment preferences (10 - 17). An additional aspect of PPC involves families as they are the ones who see the child’s discomfort and distress before death, must make difficult choices near and after the point of death, and lose a child and have to experience the sort of grief that is considered the most intense bereavement (18, 19). Reports demonstrate that the incidence of emotional disturbances is high in these families (about 50% in at least one family member). Moreover, parental grief has been reported to last deeply for 4 years. Thoughts of suicide, self-accusation and social withdrawal have also been observed in parents who have lost a child (20 - 24). Previous investigations have shown that families of dying children need respect and a true relationship provided by the nursing team; in addition, they expect health care providers to treat their children as individual patients with explicit diagnoses, relieve their distress, and give them sufficient care (25). Determining the suitable time to open a conversation about a child’s death is challenging, as the beliefs and situations of parents must be well understood. Alleviating the families’ feeling of bereavement after the loss could be achieved by multiple means available through palliative care, including psychologists and psychiatrists, clergymen and spiritual aids, and support groups (26 - 29). Former studies also indicate that health-care providers’ writing condolence messages and attending the memorial service could help families cope with the grief (26 - 29). Students and residents normally feel awkward in their confrontations with dying patients. They see death as a medical failure and generally do not perceive palliative care as an obvious component of medicine (30, 31). However, educationalists and legislators are paying more attention to the issue of palliative care in order to develop knowledge and investigation in this field (30, 31). The present study examined pediatricians’ perspectives on ethical subjects related to this field to improve the agenda of palliative care by using a questionnaire covering issues such as pediatricians’ uncluttered explanations, decision-making and psycho/spiritual support. The study targeted residents and attendings to find out if they had established maladaptive attitudes or practices.

## Method

Population and Data Collection

The present study was performed on 48 of the 92 pediatric residents, fellows and attendings of Namazi Teaching Hospital in Shiraz University of Medical Sciences, Shiraz, Iran between December 21, 2016 and May 21, 2017. The population consisted of 8 attendings, 6 fellows and 34 residents (54.2% male and 45.8% female), including 12 first-year, 13 second-year, and 9 third-year residents.

The questionnaire was designed based on previously published surveys and consultations with experts in the field of medical ethics (32 - 34). In order to evaluate its validity and reliability, the questionnaire was given to 20 residents who were selected randomly. After a month, the questionnaire was given to the same people, and the outcome was evaluated by using Spearman correlation test. Using Cronbach’s alpha ( 0.7), the questions that were found to create a significant bias were omitted (Appendix 1); moreover, Cronbach’s alpha was calculated for all questions jointly to estimate the consistency of the questionnaire, which was 0.815, and thus the questionnaire was finalized (Appendix 2). 

The questionnaire consisted of 3 sections. The first section contained the basic demographic data. The second section included queries designed in 5-point Likert-scale questions to assess the level of satisfaction with palliative care services (5 = completely satisfactory to 1 = completely unsatisfactory). The third section was based on Likert-scale responses to assess the participants’ general opinion about the topic and ethical challenges to implementation of pediatric palliative care.

The second and third section of the questionnaire consisted of 66 items and were given to participants who were gathered in a conference room on the same day. The data were extracted by a trained person who was unaware of the names and degrees of the people who filled the forms. After analysis of the data, questions that had a significant bias according to the correlation test were omitted. The finalized data were sent for statistical evaluations, and the study protocol was approved by the Medical Ethics Committee of Shiraz University of Medical Sciences, Shiraz, Iran. Participation in this research was voluntary, and participants were assured that their information would be kept confidential.

Data Management and Statistical Analysis

The collected data were reviewed for accuracy and verified by two independent experts. Descriptive statistics were analyzed by mean SD and percentage calculations. The discrete and ordinal data were compared using student’s t-test, and Spearman’s correlation and Pearson’s correlation tests were applied. The differences with a P-value 0.05 were regarded as statistically significant. Statistical analyses were performed using SPSS version 19.0 (SPSS Inc., Chicago, IL).

## Results

The data are presented in Tables 1 to 5 below. Table 1 shows the demographic data of the participants. 

The participants’ experiences (both as contributors and as observers) regarding working hours, knowledge and their exposure to palliative care are demonstrated in Table 2.

**Table 1:** Demographic data of the study participants

**Gender**	Percent
Male Female	54.2 45.8
**Marital Status**	
Single Married	70.8 29.2
**Age**	
20-29 30-39 40-49 50-60	27.1 50 18.8 4.2
**Position**	
Attending 1st year resident 2^nd^ year resident 3^rd^ year resident Fellow	16.7 25 27.1 18.8 12.5

**Table 2:** Participants’ experiences of palliative care

Months of working in pediatrics ward	53.88 70.58	
Months of working in pediatrics ICU	14.31 20.42	
Practicing hours per week	64.64 30.50	
Practicing palliative care		
Yes No	75 22.9	
Information about palliative care		
Yes No	56.3 43.8	
Source of information		
Medical education resources Internet and journals Methodological education	37.5 12.5	
	>6 hours <6 hours	10.4 52.1
Number of participants patients who needed palliative care		
1-5 6-10 11-15 16-20 >20	29.2 14.6 8.3 8.3 37.5	
Number of the participants patients who died		
1-5 6-10 11-15 16-20 >20	35.4 16.7 10.4 2.1 31.3	

Seventy-five percent of the participants reported involvement in pediatric palliative care, and 38% of the residents, fellows and attendings had had over 20 patients in need of palliative care. An additional 31% had observed more than 20 patients expiring, while 56% of the participants did not acknowledge any information about palliative care. 

Table 3 presents participants’ responses to the questions designed to assess the level of satisfaction with palliative care services. More than half of the participants (84.5% of all medical service providers and 68.9% of those in the ICU) perceived the pediatric palliative care services in Namazi Hospital, Shiraz, Iran as somewhat or completely satisfactory. 

**Table 3:** Participant’s satisfaction with palliative care services

Questionnaire Item	N	Minimum	Maximum	Mean	SD
**How do you evaluate the facilities prepared by all ** **medical provider sections for dying patients?**	45	1.00	4.00	3.0889	.76343
**How do you evaluate the appreciation of medical ** **services by families of dying patients?**	45	1.00	4.00	2.8222	.77720
**How do you evaluate the pediatric palliative care in ** **Intensive Care Units?**	45	2.00	5.00	3.4444	.78496
**How do you evaluate your own implementation of ** **pediatric palliative care?**	44	1.00	4.00	2.9318	1.02066

*Values based on Likert-scale responses (1= Completely unsatisfactory, 2= Unsatisfactory, 3= Somewhat satisfactory, 4= Satisfactory, 5= Completely satisfactory)*

**Table 4:** General comments on implementation of pediatric palliative care

**Questionnaire Item**	**N**	**Minimum**	**Maximum**	**Mean**	**SD**
The lack of education in physicians about pediatric palliative care limits appropriate services.	48	1.00	5.00	3.6875	1.15143
I have difficulty understanding the principles of palliative care.	43	1.00	5.00	3.5814	1.00552
There is a lack of information and resources in the field of palliative care.	44	1.00	5.00	3.4091	1.08517
More studies and investigations must be conducted in this field in order to enhance the application of suitable palliative care.	44	2.00	5.00	4.2273	.91152
I believe authorized education must be promoted in this field, and I will participate in this education.	43	2.00	5.00	4.2791	.90831
We must actively participate in the field of palliative care during our education.	46	2.00	5.00	3.8043	.93380
I control the expiring patients’ symptoms (such as nausea, vomiting, constipation) sufficiently.	46	1.00	5.00	4.2391	1.03676
I can identify the poor-prognosis symptoms in dying patients.	45	1.00	5.00	3.6000	1.09545
Due to lack of time and workforce, it is not possible to provide patients and their families with psychological support.	46	1.00	5.00	2.6522	1.47900
Due to cultural differences between families and healthcare providers, psychological support is unachievable.	46	1.00	5.00	3.1957	1.27575
Lessening the dying patients’ physical symptoms is the most important challenge in palliative care.	46	1.00	5.00	3.1739	.90196
Providing the physiological needs of terminal patients must be the first priority in palliative care.	45	2.00	5.00	3.7111	1.01404
Palliative care should be practiced by anesthesiologists.	44	1.00	5.00	2.2955	1.15294
Palliative care should be practiced by oncologists.	44	1.00	5.00	2.1364	1.11211
Specialists should be trained in the field of palliative care.	44	1.00	5.00	3.5000	1.19105
Palliative care should be provided in primary care facilities.	43	1.00	5.00	2.4884	1.26061
There should be units that are specialized in palliative care.	25	2.00	5.00	4.0400	.97809
Palliative care must be provided in patients’ homes.	26	2.00	5.00	3.6538	1.01754
The best way to train in this field is to capture the attendings’ experiences and bedside teachings.	44	1.00	5.00	3.5682	1.08687
The best way to train in this field is to experience the situation individually.	44	1.00	5.00	2.6591	1.19967
The best way to train in this field is to participate in related conferences.	44	1.00	5.00	3.2273	.91152
Interdisciplinary groups must be appointed between different factions of the health-care system to improve palliative care.	44	3.00	5.00	4.2045	.76492
Educating patients and their families is necessary for the improvement of palliative care.	44	2.00	5.00	4.0682	.89955

*Values based on Likert-scale responses (1= Disagree, 2= Unsure, 3= Somewhat agree, 4= Mostly agree, 5= Totally agree)*

**Table 5:** General topics in PPC

Questionnaire Item	N	Minimum	Maximum	Mean	SD
**Patients’ relatives obtain information from ** **unauthorized individuals instead of their cognizant ** **healthcare providers.**	46	1.00	5.00	4.4130	.97925
**Families have difficulty deciding on continuation of ** **invasive treatments.**	47	1.00	5.00	2.9787	1.34309
**The lack of suitable facilities and plots limits dying ** **patients and their families’ privacy. **	47	1.00	5.00	3.9787	1.29362
**The nursing team’s lack of education about pediatric ** **palliative care limits provision of appropriate services.**	46	1.00	5.00	3.5652	1.55852
**The lack of applicable guidelines about approaching ** **dying children limits proper pediatric palliative care.**	44	1.00	5.00	3.6818	1.44307

*Values based on Likert-scale responses (1= Disagree, 2= Unsure, 3= Somewhat agree, 4= Mostly agree, 5= Totally agree)*

**Table 6:** Ethical challenges of pediatric palliative care

**Questionnaire Item**	**N**	**Minimum**	**Maximum**	**Mean**	**SD**
It is very difficult for me to discuss palliative care with patients and their families.	28	1.00	5.00	3.5714	1.31736
I provide the information to dying patients and their families about altering curative treatments to palliative therapy.	47	1.00	5.00	3.5106	1.26615
I announce the diagnosis of the life-threatening disease to patients’ families in a meeting.	47	1.00	5.00	3.9149	1.21279
I ask the dying children to participate in these meetings.	47	1.00	4.00	1.6170	.89814
I explain the DNR^1^ protocol to patients’ families.	47	1.00	5.00	2.5319	1.48691
Families have difficulty deciding on the DNR protocol or termination of mechanical ventilation.	47	1.00	5.00	4.0213	1.15136
Patients’ families are not prepared to accept that their child’s disease is incurable.	47	1.00	5.00	3.6596	1.08901
I am afraid of discussing the cessation of curative treatments.	46	1.00	5.00	2.6522	1.32023
It is very challenging for me to discuss altering curative treatments to palliative therapy with patients and their families.	47	1.00	5.00	3.6809	1.14410
I feel awkward facing the families’ reactions, and I do not know what to tell them.	46	1.00	5.00	3.5000	1.11056
Patients’ families do not understand the terminal nature of the disease, so I do not explain the situation to them.	47	1.00	5.00	1.8723	1.22682
Altering curative treatments to palliative care could hurt the families’ trust in the health-care system that did not cure their child’s disease.	47	1.00	5.00	2.7660	1.25478
Patients’ families must decide whether to start palliative care or not.	47	1.00	5.00	4.0638	1.05097
Patients’ families must be informed after the decision has been made to start palliative care.	46	1.00	5.00	3.2609	1.59770
I assist the patients and their families with spiritual and psychological support.	47	1.00	5.00	4.4043	.87625
Initiation of palliative care resembles doing nothing for the patients.	46	1.00	4.00	2.0000	1.15470
Palliative care has been designed to decrease the economic burden of dying patients.	45	1.00	5.00	1.9778	1.19637
Continuation of curative treatment in dying patients sometimes seems illogical.	45	1.00	5.00	3.1111	1.30074
In patients with life-threatening conditions, early initiation of palliative care could be more beneficial.	46	1.00	5.00	3.4565	1.14904
Even if there is no hope for treatment of the disease, we must continue the curative and invasive therapies.	45	1.00	5.00	2.0222	1.17722
We must continue invasive treatments for dying patients because we could be sued by their families.	45	1.00	5.00	3.0889	1.44320
Doctors do not accept their patients are dying.	46	1.00	5.00	2.0000	1.21106
We must discuss the situation of every expiring patient uniquely, considering their conditions.	42	1.00	5.00	4.3333	1.00406
No matter how long I have practiced in the field of pediatric palliative care, encountering children who are suffering from end of life conditions is difficult for me.	46	1.00	5.00	3.1522	1.42933
Doctors have a significant role in supporting the patients and their families through psychological and spiritual help.	46	1.00	5.00	4.4130	.83203
Psychological and spiritual support must be provided by other groups such as psychologists and nurses.	46	1.00	5.00	2.9783	1.34146
Providing the patients and their families with appropriate information is the greatest challenge.	45	2.00	5.00	3.9111	1.04059
Providing psychological and spiritual support is the most important challenge in this field.	46	2.00	5.00	4.0217	.95427
Palliative care services are important in dealing with patients.	44	2.00	5.00	4.2273	.96119
How often do you encounter ethical issues with regard to pediatric palliative care?	45	1.00	5.00	3.8444	1.14724

*Values are based on Likert-scale responses (1= Disagree, 2= Unsure, 3= Somewhat agree, 4= Mostly agree, 5= Totally agree); 1= Never, 2= 1 time until now, 3= 2-3 times until now, 4= Once a week, 5= Always*

## Discussion

There are many challenges to providing decent palliative care for children such as symptom controlling problems in terms of scheduling, sufficiency, and competency of management. Some of these challenges could be due to fear of speeding the child’s death, difficulties associated with management of neonates, the shift from curative care to end of life care, noticing the issue of PPC at a national level, personal differences among different patients, background dissimilarities of patients, lack of time, financial restrictions, dealing with patients’ apprehensions, and acceptance of death by patients, families and health-care providers (5, 6, 35 - 38). On the other hand, there is inadequate literature on the essentials and effectiveness of many PPC interventions which could be due to absence of study cases, unavailability of a baseline score for assessment of pain and QoL in pediatrics, and the fact that PPC is a novel subject (39). Moreover, there is no evidence-based tool to determine which patients would benefit from palliative care, and therefore initiation of palliative care should be personalized (38).

Previous studies have suggested policies with regard to PC for various psychosomatic symptoms such as dyspnea, pain, nausea, seizures, agitation, anxiety, depression and grief, which can develop differently from one person to another (40 - 45). However, palliative care organizations have been mostly focused on physical care and the medical treatment of suffering rather than the psychological, sociological and spiritual aspects of death. Studies have shown that health-care professionals could be frustrated due to compassion fatigue and burnout while providing PPC. This might be due to interaction problems, disagreements on decisions, lack of system support such as excessive workload and workforce unavailability, subjugated grief, and legal issues (6). Palliative care practitioners have reported a feeling of “powerlessness” over dying patients, and that “there is always something more to be done”; thus the pressure of choosing between acting or not acting arises in daily communications with patients as well as in the philosophy of good death (46). 

Most of our participants (75%) claimed that they practiced PPC but about half of them stated that they did not have adequate information about PPC. Most of the applicants who asserted they had received training in PPC said they had obtained information in this field from medical education resources. Most of the participants evaluated their satisfaction with palliative care services as somewhat satisfactory; however, they were mostly not pleased with the appreciation of medical services by families of dying patients. Our data also revealed that improper palliative care services may be due to a number of reasons such as lack of education in physicians and nurses, insufficiency of educational courses and workshops about PPC, problems in identification of poor-prognosis symptoms, inadequate understanding of the principles of PPC, lack of resources, shortage of research in PPC, lack of time and workforce, cultural differences between health-care providers and patients’ families, absence of a systematic approach and role modeling around PPC, and insufficient family education. Our applicants also declared that some problems could be related to the families of dying patients, for instance they might have difficulty deciding on the continuation of treatments, or attempt to obtain information from unauthorized persons. 

Although most of our applicants admitted to some difficulties in starting palliative care discussions, they said that they mostly informed patients’ families about the situation, but they did not involve the children in educational meetings. 

Insufficient education both in families and health-care providers may also lead to certain misunderstandings about PPC. The consequences may include: failure to recognize the terminal condition of the disease, emotional complications, inconsistencies in terms of rituals and spiritual beliefs, being sued by the dying children’s families, and impairment of the families’ trust in the health-care system.

Most of the participants insisted that the families must be the ones to make the decision to start palliative care; however, informing them after deciding to start PPC was also perceived as favorable. Our results also depicted that although physicians mostly provided psychological and spiritual support to the families of dying children, they generally preferred the task to be left to other groups such as nurses and psychologists.

Even though decreasing the economic burden of dying patients is a major concern in PPC, it did not appear to be particularly important for our participants. On the other hand, our physicians mostly insisted on the effectiveness and early initiation of PPC in dying patients in order to reduce suffering and end-of-life complications for these patients.

Our participants also perceived psychological and spiritual support to be the most important challenges to pediatric palliative care; thus, further investigations are required in order to provide comprehensive guidelines in this field.

## Conclusion

Of the numerous issues that could be considered as challenges to the implementation of PPC mentioned above, the spiritual and psychological aspects of PPC were found to be the most important ones. The results showed that it is essential to educate patients’ families as well as health-care providers, and that educational courses and obligatory guidelines would be helpful in this case. Investigations are still highly required to determine other demands and considerations regarding provision of a comprehensive guideline on PPC. 

## Appendix

**Appendix 1:** Evaluation of validity and the reliability of the questionnaires by using Cronbach’s alpha test

**Question No.**	**Cronbach’s Alpha**
1	0.877
2	1
3	1
4	0.954
5	1
6	0.926
7	0.993
8	0.962
9	0.963
10	1
11	0.953
12	1
13	1
14	1
15	1
16	0.884
17	1
18	0.985
19	1
20	0.640
21	0.802
22	0.964
23	0.883
24	0.768
25	0.808
26	0.889
27	0.982
28	0.958
29	0.985
30	0.986
31	0.864
32	0.960
33	1
34	0.859
35	0.924
36	0.924
37	0.673
38	0.734
39	0.628
40	0.728
41	0.803
42	0.304
43	0.784
44	0.829
45	0.881
46	0.611
47	0.968
48	0.931
49	1
50	0.951
51	0.961
52	1
53	1
54	0.974
55	1
56	0.909
57	0.980
58	0.970
59	0.975
60	0.689
61	0.988
62	1
63	0.898
64	0.961
65	0.758
66	0.941

**Appendix 2:** The finalized questionnaire evaluating participants’ satisfaction with palliative care services

**Items**	**Completely ** **satisfactory = 5**	**Satisfactory = ** **4**		**Somewhat ** **satisfactory = ** **3**			**Unsatisfactory = ** **2**		**Completely ** **unsatisfactory** **= 1**	
How do you evaluate the facilities prepared by all medical provider sections for dying patients?	Completely satisfactory	Satisfactory		Somewhat satisfactory			Unsatisfactory		Completely unsatisfactory	
How do you evaluate the appreciation of medical services by families of dying patients?	Completely satisfactory	Satisfactory		Somewhat satisfactory			Unsatisfactory		Completely unsatisfactory	
How do you evaluate the pediatric palliative care in Intensive Care Units?	Completely satisfactory	Satisfactory		Somewhat satisfactory			Unsatisfactory		Completely unsatisfactory	
How do you evaluate yourself implementing pediatric palliative care?	Completely satisfactory	Satisfactory		Somewhat satisfactory			Unsatisfactory		Completely unsatisfactory	
**General topics and ethical challenges to implementation of pediatric palliative care**										
How many times do you encounter ethical issues with regard to pediatric palliative care?			Always		Once a week	2 - 3 times until now		1 time until now		Never
Patients’ relatives obtain information from unauthorized individuals instead of their cognizant healthcare providers.			Always		Once a week	2 - 3 times until now		1 time until now		Never
Families have difficulty deciding on continuation of invasive treatments.			Always		Once a	2 - 3 times		1 time		Never
The lack of suitable facilities and plots limits dying patients and their families’ privacy.			Always		Once a	2 - 3 times		1 time		Never
The nursing team’s lack of education about pediatric palliative care limits provision of appropriate services.			Always		Once a week	2 - 3 times until now		1 time until now		Never
The lack of applicable guidelines about approaching dying children limits proper pediatric palliative care.			Always		Once a week	2 - 3 times until now		1 time until now		Never
It seems very difficult to me to start a discussion about palliative care.			Totally agree		Mostly agree	Somewhat agree		Unsure		Disagree
Physicians’ lack of education about pediatric palliative care limits provision of appropriate services.			Totally agree		Mostly agree	Somewhat agree		Unsure		Disagree
I provide the information to dying patients and their families about altering curative treatments to palliative therapy.			Totally agree		Mostly agree	Somewhat agree		Unsure		Disagree
I announce the diagnosis of the life-threatening disease in a meeting to patients’ families.			Totally agree		Mostly agree	Somewhat agree		Unsure		Disagree
I ask the dying children to participate in these meetings.			Totally agree		Mostly agree	Somewhat agree		Unsure		Disagree
I explain the DNR protocol to the patients’ families.			Totally agree		Mostly agree	Somewhat agree		Unsure		Disagree
Patients’ families are not prepared to accept that their child’s disease is incurable.			Totally agree		Mostly agree	Somewhat agree		Unsure		Disagree
It is very challenging for me to discuss altering curative treatments to palliative therapy with patients and their families.			Totally agree		Mostly agree	Somewhat agree		Unsure		Disagree
I feel awkward facing the families’ reactions, and I do not know what to tell them.			Totally agree		Mostly agree	Somewhat agree		Unsure		Disagree
Patients’ families do not understand the terminal nature of the disease, so I do not explain the situation to them.			Totally agree		Mostly agree	Somewhat agree		Unsure		Disagree
Altering curative treatments to palliative care could hurt the families’ trust in the health-care system that did not cure their child’s disease.			Totally agree		Mostly agree	Somewhat agree		Unsure		Disagree
Patients’ families must decide whether to start palliative care or not.			Totally agree		Mostly agree	Somewhat agree		Unsure		Disagree
Patients’ families must be informed after the decision has been made to start palliative care.			Totally agree		Mostly agree	Somewhat agree		Unsure		Disagree
I am afraid of discussing the cessation of curative treatments.			Totally agree		Mostly agree	Somewhat agree		Unsure		Disagree
Families have difficulty deciding on the DNR protocol or termination of mechanical ventilation.			Totally agree		Mostly agree	Somewhat agree		Unsure		Disagree
I assist the patients and their families with spiritual and psychological support.			Totally agree		Mostly agree	Somewhat agree		Unsure		Disagree
I control the expiring patient’s symptoms (such as nausea, vomiting, constipation) sufficiently.			Totally agree		Mostly agree	Somewhat agree		Unsure		Disagree
I can identify the poor-prognosis symptoms in dying patients.			Totally agree		Mostly agree	Somewhat agree		Unsure		Disagree
Initiation of palliative care resembles doing nothing for the patients.			Totally agree		Mostly agree	Somewhat agree		Unsure		Disagree
Palliative care has been designed to decrease the economic burden of dying patients.			Totally agree		Mostly agree	Somewhat agree		Unsure		Disagree
Continuation of curative treatments in dying patients seems illogical sometimes.			Totally agree		Mostly agree	Somewhat agree		Unsure		Disagree
In patients with life-threatening conditions, early initiation of palliative care could be more beneficial.			Totally agree		Mostly agree	Somewhat agree		Unsure		Disagree
Even if there is no hope for treatment of the disease, we must continue the curative and invasive therapies.			Totally agree		Mostly agree	Somewhat agree		Unsure		Disagree
We must continue invasive treatments for dying patients because we could be sued by their families.			Totally agree		Mostly agree	Somewhat agree		Unsure		Disagree
Doctors do not accept their patients are dying.			Totally agree		Mostly agree	Somewhat agree		Unsure		Disagree
We must discuss the situation of every expiring patient uniquely, considering their conditions.			Totally agree		Mostly agree	Somewhat agree		Unsure		Disagree
No matter how long I have practiced in the field of pediatric palliative care, encountering children who are suffering from end of life conditions is difficult for me.			Totally agree		Mostly agree	Somewhat agree		Unsure		Disagree
We must actively participate in the field of palliative care during our education.			Totally agree		Mostly agree	Somewhat agree		Unsure		Disagree
Doctors have a significant role in supporting the patients and their families through psychological and spiritual help.			Totally agree		Mostly agree	Somewhat agree		Unsure		Disagree
Psychological and spiritual support must be provided by other groups such as psychologists and nurses.			Totally agree		Mostly agree	Somewhat agree		Unsure		Disagree
Due to lack of time and workforce, it is not possible to provide patients and their families with psychological support.			Totally agree		Mostly agree	Somewhat agree		Unsure		Disagree
Due to cultural differences between families and healthcare providers, psychological support is unachievable.			Totally agree		Mostly agree	Somewhat agree		Unsure		Disagree
Lessening the dying patients’ physical symptoms is the most important challenge in palliative care.			Totally agree		Mostly agree	Somewhat agree		Unsure		Disagree
Providing the physiological needs of terminal patients must be the first priority in palliative care.			Totally agree		Mostly agree	Somewhat agree		Unsure		Disagree
Providing the patients and their families with appropriate information is the greatest challenge.			Totally agree		Mostly agree	Somewhat agree		Unsure		Disagree
Providing psychological and spiritual support is the most important challenge in this field.			Totally agree		Mostly agree	Somewhat agree		Unsure		Disagree
**Optional Questions:**										
Palliative care services are important in dealing with patients.			Totally agree		Mostly agree	Somewhat agree		Unsure		Disagree
I have difficulty understanding the principles of palliative care.			Totally agree		Mostly agree	Somewhat agree		Unsure		Disagree
There is a lack of information and resources in palliative care.			Totally agree		Mostly agree	Somewhat agree		Unsure		Disagree
Palliative care should be practiced by anesthesiologists.			Totally agree		Mostly agree	Somewhat agree		Unsure		Disagree
Palliative care should be practiced by oncologists.			Totally agree		Mostly agree	Somewhat agree		Unsure		Disagree
Specialists should be trained in palliative care.			Totally agree		Mostly agree	Somewhat agree		Unsure		Disagree
Palliative care should be provided in primary care facilities.			Totally agree		Mostly agree	Somewhat agree		Unsure		Disagree
There should be units that are specialized in palliative care.			Totally agree		Mostly agree	Somewhat agree		Unsure		Disagree
Palliative care must be provided in patients’ homes.			Totally agree		Mostly agree	Somewhat agree		Unsure		Disagree
I believe authorized education must be promoted in this field, and I will participate in this education.			Totally agree		Mostly agree	Somewhat agree		Unsure		Disagree
The best way to train in this field is to capture the attendings’ experiences and bedside teachings.			Totally agree		Mostly agree	Somewhat agree		Unsure		Disagree
The best way to train in this field is to experience the situation individually.			Totally agree		Mostly agree	Somewhat agree		Unsure		Disagree
The best way to train in this field is to participate in related conferences.			Totally agree		Mostly agree	Somewhat agree		Unsure		Disagree
Interdisciplinary groups must be appointed between different factions of the health-care system to improve palliative care.			Totally agree		Mostly agree	Somewhat agree		Unsure		Disagree
Educating patients and their families is necessary for the improvement of palliative care.			Totally agree		Mostly agree	Somewhat agree		Unsure		Disagree
More studies and investigations must be conducted in this field in order to enhance the application of suitable palliative care.			Totally agree		Mostly agree	Somewhat agree		Unsure		Disagree
